# Endogenous Purulent Pericarditis Due to Klebsiella aerogenes in a Patient With Traumatic Chest Injury: A Case Report

**DOI:** 10.7759/cureus.52378

**Published:** 2024-01-16

**Authors:** Gi Eun Kim, Soubiya Ansari, Gabriala N Andrews, Sreethish Sasi, Jouhar Kolleri, Tasneem A Abdallah, Ibrahim F Hassan, Muna Al Maslamani

**Affiliations:** 1 Internal Medicine, Hamad General Hospital, Doha, QAT; 2 Infectious Diseases, Hamad Medical Corporation, Doha, QAT; 3 Radiology, Hamad Medical Corporation, Doha, QAT; 4 Critical Care Medicine, Hamad General Hospital, Doha, QAT

**Keywords:** multidrug resistant, meropenem, pericardiocentesis, klebsiella aerogenes, purulent pericarditis

## Abstract

Purulent pericarditis is a rare but serious medical condition caused by an infection that spreads to the pericardial space surrounding the heart. Gram-positive organisms are the most common pathogens associated with purulent pericarditis. However, there has been a shift in recent years toward gram-negative bacteria. *Klebsiella aerogenes* is a rare pathogen that has never been linked to purulent pericarditis. In this report, we describe the case of a 40-year-old male patient with chronic bronchiectasis who, two months after suffering an injury, developed purulent pericarditis due to an uncommon organism, *K. aerogenes*. During his stay in the hospital, the patient developed several infections caused by *K. aerogenes.* These included bacteremia and ventilator-associated pneumonia (VAP). Beta-lactamase-inducible *K. aerogenes* was grown in pericardial fluid culture following an emergency pericardiocentesis. The organism was resistant to carbapenems in a sputum culture, even though it was sensitive to meropenem in a blood culture. The patient had hypotension, requiring inotropes, and continued persistent bacteremia due to *K. aerogenes*. The patient had a heart attack with no pulse or electrical activity and died despite getting the best care possible. In light of this example, it is crucial to think about *K. aerogenes* and other rare organisms as possible pathogens in purulent pericarditis, especially in people who do not normally have known risk factors for this condition. Multidrug resistance patterns can make treatment more complicated, and aggressive care may be necessary in critically ill patients with chronic bacteremia.

## Introduction

Purulent pericarditis is a rare but serious medical condition caused by an infection that spreads to the pericardial space surrounding the heart, causing inflammation and pus accumulation. Numerous pathogens, including bacteria, viruses, and fungi, can cause this condition, leading to complications such as sepsis, cardiac tamponade, and death [1]. Gram-positive organisms, including *Streptococcus* and *Staphylococcus* species, have traditionally been the most common pathogens linked to purulent pericarditis [1]. However, a noticeable trend has emerged toward gram-negative bacteria, such as *Proteus, Escherichia coli, Pseudomonas*, and occasionally *Haemophilus influenzae *and *Klebsiella pneumoniae *[2]. This shift may be attributed to the increased utilization of broad-spectrum antibiotics, potentially leading to the emergence of resistant strains of gram-negative bacteria [2]. Furthermore, invasive medical procedures such as dialysis and thoracic surgery can increase the risk of gram-negative infections [3]. *Klebsiella aerogenes*, previously known as *Enterobacter aerogenes,* is a gram-negative bacterium that has been rarely linked to purulent pericarditis. This pathogen, however, has been frequently linked to hospital-acquired infections, including urinary tract infections, pneumonia, and sepsis [4,5]. This case report emphasizes the importance of considering rare pathogens as a possible cause of purulent pericarditis, particularly in patients with a history of hospitalization or who have undergone invasive medical procedures. Early detection and treatment are critical for avoiding serious complications and improving patient outcomes.

## Case presentation

A 40-year-old male was admitted to the hospital following a car accident. He was known to have chronic bronchiectasis and was on regular steroid inhalers and bronchodilators when needed. He had no other comorbidities and was not on any other home medications. He had a 25-pack-year smoking history and reported consuming excessive amounts of alcohol on a daily basis for the past 10 years. There was no history of any significant illness in the family.

There was a penetrating injury to the anterior left chest at the level of the second and third intercostal spaces. The wound was actively bleeding. Emergency medical services (EMS) performed the dressing and thoracostomy insertion. The EMS team attempted to intubate the patient but was unsuccessful, so he was placed on a laryngeal mask airway. Upon arrival at the hospital, he was intubated and found to have left chest trauma caused by a foreign metallic object, resulting in a bleeding upper left chest sucking wound. Foreign body removal necessitated an emergency thoracotomy, and two chest tubes were inserted to drain the hemothorax. As a result of his severe respiratory failure and underlying bronchiectasis, he was placed on a veno-venous extracorporeal membrane oxygenation (VV-ECMO). He was then admitted to the medical intensive care unit on VV-ECMO. He remained critically ill and required ventilatory and cardiovascular support.

Two weeks after admission, he developed acute tachypnea, tachycardia, and desaturation requiring a fraction of inspired oxygen (FiO_2_) of 60%. A septic workup was ordered, and he was empirically started on piperacillin-tazobactam, which was later escalated to meropenem because he was not clinically improving. *Klebsiella aerogenes* grew in the culture from tracheal aspirate and blood. Pan-computed tomography (CT) revealed an apical hematoma and a collapse of the lower lobe of the left lung. According to the antibiotic sensitivity test, trimethoprim/sulfamethoxazole (co-trimoxazole) replaced meropenem. His clinical status improved, and he completed two weeks of co-trimoxazole. Further, during his hospital stay, he developed multiple *K. aerogenes* infections, including ventilator-associated pneumonia (VAP) and bacteremia. He received a second round of co-trimoxazole for seven days after receiving a diagnosis of VAP and bacteremia caused by the same organism two months after admission. He continued to be critically ill on VV-ECMO, requiring ventilatory and cardiovascular support.

**Figure 1:**
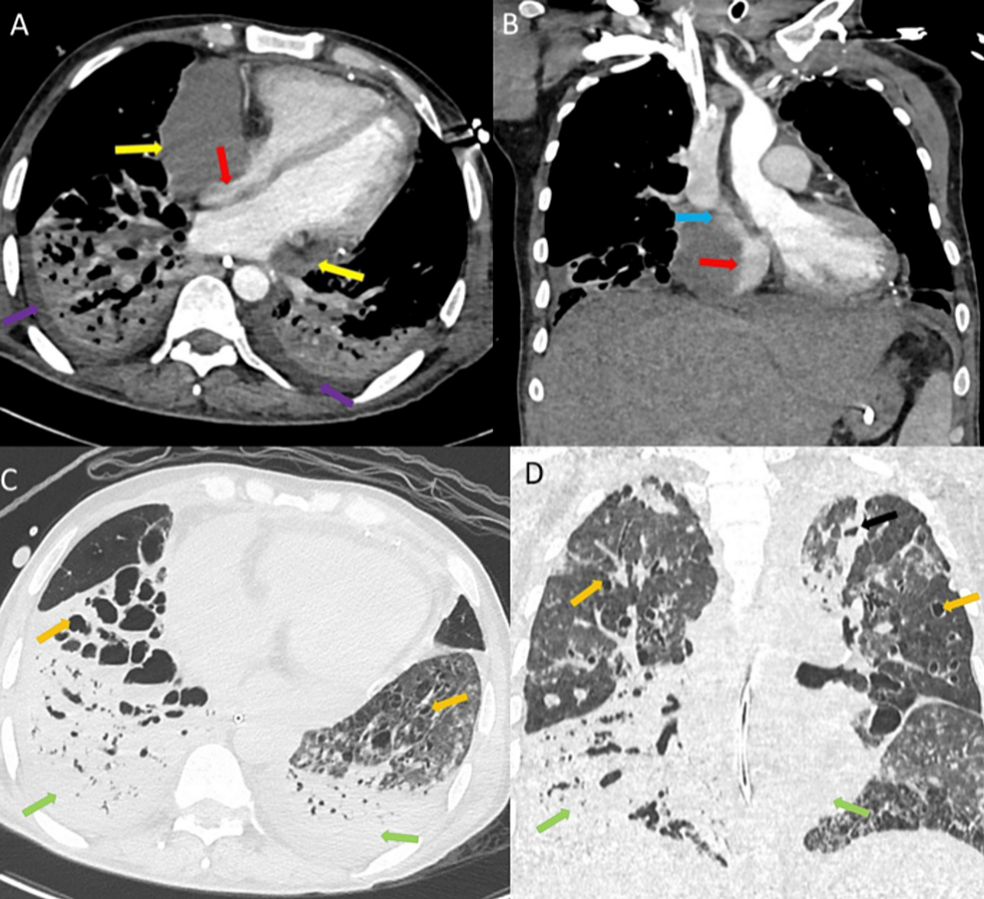
CT of the thorax with IV contrast, soft tissue window A) Axial and B) coronal cuts showing significant large pericardial effusion involving the inferior and right pericardial regions (yellow arrows) causing severe compression on the right atrium with almost total obliteration of the lumen (red arrow) and compression on the lower end of the superior vena cava (blue arrow). Lung window C) axial and D) coronal images demonstrating bilateral areas of consolidation, ground glass opacification, and centrilobular nodules in both upper lobes with consolidation in the lower lobes (green arrows); bilateral cystic bronchiectasis (orange arrows), left upper lobe irregular cavity (black arrow), and newly formed bilateral pleural effusion (purple arrows) were also seen CT: computed tomography

**Figure 2:**
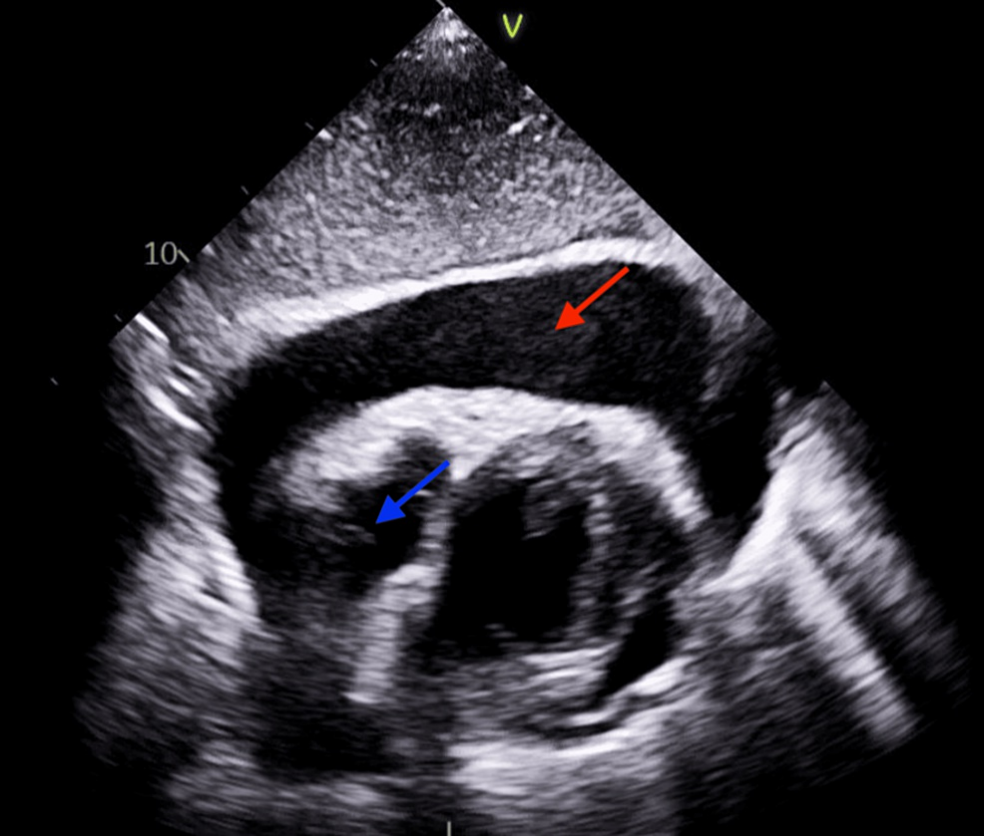
Transthoracic echocardiogram (TTE) showing pericardial effusion TTE showing large pericardial effusion (red arrow) and collapsed right atrium (blue arrow)

Two weeks later, while on ECMO, the patient's condition worsened with an increase in oxygen requirement, profound hypotension, and elevated inflammatory markers. CT of the chest (Figure 1) revealed a new bilateral pleural effusion, and a transthoracic echocardiogram (Figure 2) revealed a pericardial effusion with tamponade-like characteristics.

Cardiologists performed an emergency pericardiocentesis and drained 400 mL of purulent fluid. Three days later, a transesophageal echocardiogram revealed localized pericardial effusion causing right atrial tamponade; therefore, video-assisted thoracoscopic surgery (VATS) with pericardial drain insertion was performed to create a pericardial window. Bloody pericardial fluid containing 22,000 red blood cells (RBCs) and 11,000 white blood cells (WBCs) was drained. The culture of pericardial fluid yielded beta-lactamase-inducible *K. aerogenes*, so the antibiotic was escalated to meropenem. Simultaneously, he had the same organism growing in blood and tracheal aspirate cultures. It was sensitive to meropenem in the blood culture but resistant to carbapenems in the pericardial fluid and tracheal aspirate cultures. Therefore, he was started on a four-week course of ceftazidime-avibactam. The sensitivity patterns of the two strains of *Klebsiella aerogenes* are shown in Table 1.

**Table 1:** The sensitivity pattern of the two strains of Klebsiella aerogenes: (A) from blood and (B) from respiratory sample

Antibiotic	Isolate A (from blood)	Isolate B (from respiratory sample and pericardial fluid)
Amoxicillin-clavulanate	Resistant	Resistant
Ampicillin	Resistant	Resistant
Aztreonam	Resistant	Resistant
Cefepime	Intermediate	Resistant
Ceftazidime-avibactam	Sensitive	Sensitive
Ceftolozane-tazobactam	Sensitive	Resistant
Ceftriaxone	Resistant	Resistant
Cefuroxime	Resistant	Resistant
Colistin	Sensitive	Sensitive
Ertapenem	Sensitive	Resistant
Fosfomycin	Sensitive	Resistant
Gentamicin	Sensitive	Sensitive
Meropenem	Sensitive	Resistant
Meropenem-vaborbactam	Sensitive	Sensitive
Piperacillin-tazobactam	Resistant	Resistant
Trimethoprim-sulfamethoxazole	Sensitive	Sensitive

Two weeks after the onset of the initial cardiac tamponade, a repeat echocardiogram revealed that the pericardial effusion had decreased and the tamponade had resolved. The patient continued to have *Klebsiella aerogenes* bacteremia. A month later, he developed hypotension, necessitating inotropes. Blood and endotracheal tube aspirate cultures remained positive for *Klebsiella aerogenes*, and he was restarted on ceftazidime-avibactam. It was believed that a central-line-associated infection, or ECMO line infection, was the likely source. Seven days after resuming antibiotic treatment, neither his consciousness nor his need for inotropes improved significantly. On day 128 of ECMO, the critical care team decided, after consulting with the patient's family, to change the patient's code status to do not attempt resuscitation (DNAR) without escalation of care. Despite maximal inotropic support, the patient developed progressive hypotension and shock in the following week and died of cardiac arrest with pulseless electrical activity.

## Discussion

The patient had purulent pericarditis caused by *Klebsiella aerogenes*. This organism is almost certainly the same one that caused the patient's pneumonia, which ultimately led to pericarditis. Notably, while *Klebsiella pneumoniae* pericarditis has been reported multiple times [6-10], *Klebsiella aerogenes* pericarditis has only been rarely reported. The patient had a thoracotomy for a chest injury and an insertion of a VV-ECMO machine for acute respiratory distress syndrome (ARDS). Two months separated these events from the onset of purulent pericarditis. Seventy-nine percent of patients who develop purulent pericarditis as a complication of cardiac or thoracic surgery die within two months of the surgery, according to a review article by Bulkley et al. [11]. The majority of cases of infective pericarditis as a surgical complication occur within a few weeks following surgery. Most people who have purulent pericarditis have had the pericardial sac manipulated, which can lead to sterile adhesive pericarditis and help organisms grow if they get into the pericardial space in different ways [11].

We believe that it is unlikely that the chest injury and the surgery the patient underwent caused his purulent pericarditis. A review article comparing the pre-antibiotic and antibiotic eras found a significant increase in the incidence of purulent pericarditis in patients who had undergone cardiac or thoracic procedures and had chronic kidney disease, alcoholism, immunosuppression, or cancer, compared to young, relatively healthy patients who developed purulent pericarditis due to pulmonary infection [12]. Prior to admission, our patient had a history of alcoholism, which is consistent with the current pattern of comorbidities in patients with purulent pericarditis. He got several infections in the hospital, such as VAP and bacteremia with *K. aerogenes*. He had active VAP and bacteremia for one week before getting purulent pericarditis. Therefore, it is likely that the infection causing purulent pericarditis was present for an extended period of time elsewhere in the patient's body, most likely in the lungs.

Pericardiocentesis and pericardial fluid analysis, which reveal microscopic or macroscopically visible pus, are required to make a definitive diagnosis of purulent pericarditis [13]. Forty-two to seventy-seven percent of patients with purulent pericarditis exhibit cardiac tamponade [14]. Beck's triad of hypotension, jugular vein enlargement, and muffled heart sounds are present [15]. An echocardiogram revealed tamponade in our patient, necessitating immediate pericardiocentesis to alleviate symptoms of obstructive shock. Pericarditis can be difficult to diagnose due to the absence of typical symptoms and the frequent misattribution of fever and elevated inflammatory markers to another infection that is the source of the infection. Only 18% of patients were diagnosed with purulent pericarditis prior to death, according to a review article [12]. Moreover, because pericardiocentesis is a high-risk procedure for critically ill patients, it is not typically performed on patients with mild pericardial effusion.

## Conclusions

This report details the unusual occurrence of pyopericardium in a patient with VAP due to *K. aerogenes*. Although this patient had a history of chest trauma and cardiothoracic surgery, both of which could have contributed to the development of purulent pericarditis, the two-month delay between the trauma and the complication suggests otherwise. In addition, the rarity of *K. aerogenes* associated with purulent pericarditis emphasizes the need for physicians to have a high index of suspicion for organisms other than *S. aureus, S. pneumoniae, *and *Mycobacterium tuberculosis* in pus-producing pericardial cultures.
